# The State of the Art in Septoplasty: A Review of the Latest Achievements

**DOI:** 10.1155/bmri/7066464

**Published:** 2026-03-06

**Authors:** Masoud Janipour, Fatemeh Rezaei-Tazangi

**Affiliations:** ^1^ Otolaryngology Research Centre, Department of Otolaryngology, Shiraz University of Medical Sciences, Shiraz, Iran, sums.ac.ir; ^2^ Department of Anatomy, School of Medicine, Fasa University of Medical Sciences, Fasa, Iran, fums.ac.ir

**Keywords:** modalities, nasal septal deviation, nasal septal perforation, septoplasty

## Abstract

The deviated nasal septum is the most common cause of nasal airway obstruction, frequently diagnosed by otolaryngologists in clinical practice. Reports displayed that this condition affects 70%–80% of the population. The etiology of this upper airway problem is mainly related to intrinsic deformity or quadrangular cartilage dislocation from its bony borders, which can finally confer different disorders, such as breathing problems, sinus infections, snoring, headaches, exacerbating sleep apnea, and sleep disturbances. One of the most common methods for correcting a deviated nasal septum is septoplasty. However, this procedure still requires significant improvements to enhance its effectiveness and reduce the risk of surgery‐related complications, particularly nasal septal perforation and the recurrence of deviation in adolescents and adults. In recent years, numerous strategies have been proposed to enhance septoplasty outcomes and reduce surgery‐related complications. Many of these approaches focus on improving pain management, restoring olfactory function, alleviating symptoms, and enhancing clinical nasal outcomes. Additionally, they are aimed at promoting healing of the nasal mucosa and close septal perforations following surgery. This narrative review critically appraises the latest advancements in septoplasty, contrasting high‐level evidence from large multicenter trials with promising but preliminary findings from novel techniques aimed at enhancing outcomes and reducing complications.

## 1. Introduction

Nasal obstruction is known as one of the most prevalent symptoms observed by otolaryngologists in the clinic [1]. It can be described as a feeling of discomfort caused by inadequate airflow through the nose [2]. The etiology of unilateral nasal obstruction is multifactorial, encompassing physiological, anatomical, congenital, and acquired factors like trauma or infection. Nevertheless, a deviated nasal septum remains the primary cause [3–5]. According to reports, the prevalence of this upper airway common disease in the general population is between 70%–80% [6]. The nasal septum is a supportive structure in the midline of the nasal cavity that not only has a key role in maintaining the shape and architecture of the nose but also aids in modulating respiration and airflow [4]. The nasal septum, one of the most critical components of the facial skeletal structure, comprises the septal cartilage, the vomer bone, and the perpendicular plate of the ethmoid bone [7]. This cartilaginous element keeps in touch with other structures, like the frontal bone, nasal bone, maxillary bone, cribriform plate, sphenoid bone, and palatine bone [8]. Regarding bone evolution, two significant growth peaks have been determined for the nasal septum in the first 2 years following birth and during juvenility due to the nose and nasal septal growth until approximately the age of 16–17 [9]. A deviated nasal septum can cause breathing problems, sinus infections, and continual nosebleeds, as well as snoring, headaches, exacerbating sleep apnea, and sleep disturbances [10]. This condition can stem from quadrangular cartilage dislocation from its bony borders or as a result of an intrinsic deformity that affects the perpendicular plate of the ethmoid, vomer bone, and quadrilateral cartilage [11]. Fortunately, surgery (septoplasty) has commonly been utilized to correct deviated nasal septal at various locations [12]. However, it has been mentioned that the reemergence of deviation in adolescent and adult cases of deviated nasal septum undergoing surgery is possible [13]. The significant growth difference between the midfacial skeleton and nasal septal cartilage may give rise to redeviation, which restricts surgical techniques in adolescents [13]. Therefore, there is still an unmet need to offer more effective septoplasty‐related approaches to minimize the adverse effects of the surgery and risk of redeviation in afflicted patients. Herewith, we are aimed at reviewing the latest achievements in septoplasty‐related strategies in recent years for patients with deviated nasal septum to help otolaryngologists and clinicians better manage subjects with deviated nasal septum in clinical practice.

## 2. Anatomy of the Nasal Cavity

**Figure 1:**
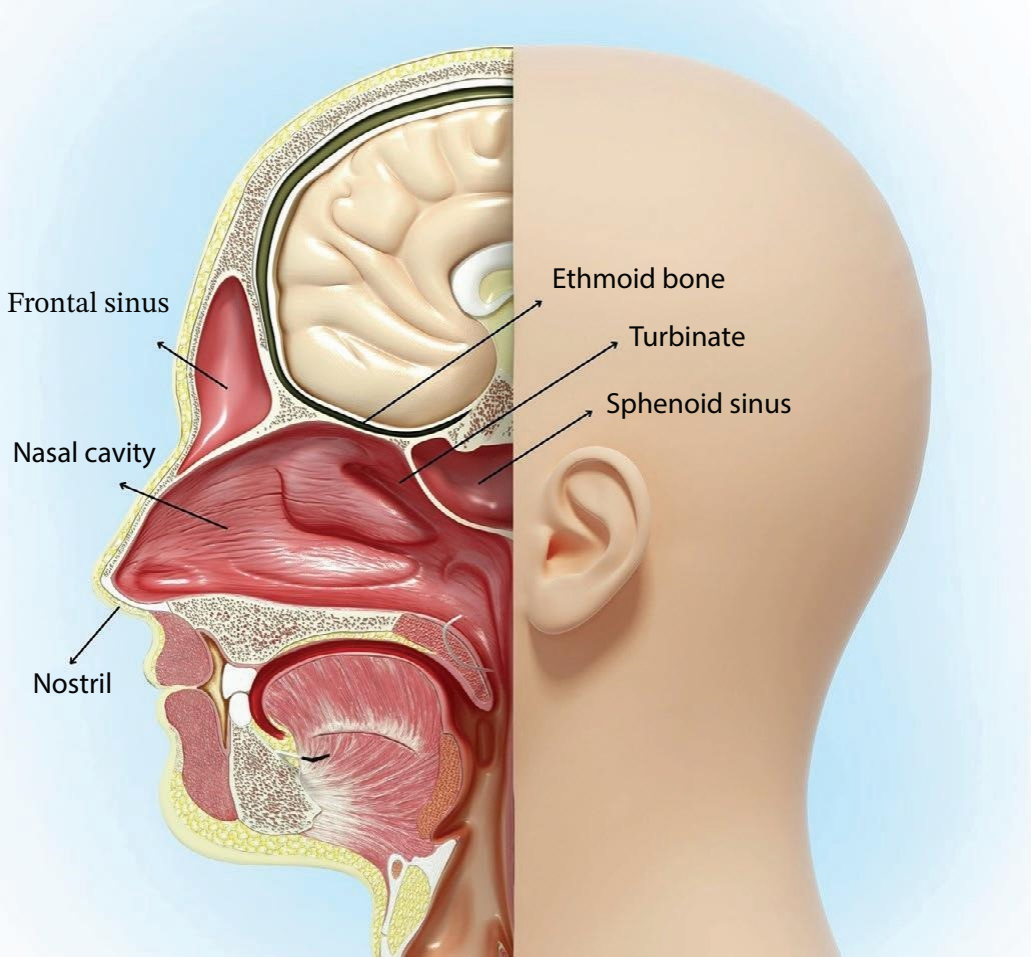
A cross section of throat and nose anatomy.

The nasal septum is placed in the middle of the nasal cavity and divides it into right and left parts [14]. This septum constructs the medial wall of the nasal cavity [15]. The lateral wall of the cavity possesses nasal turbinates or conchae (Figure 1), divided into inferior, superior, and middle parts, allowing for heating, filtration, and humidification of the breathing air [7]. The posterior section of the cavity consists of the perpendicular plate of the ethmoid bone and the vomer bone, whereas the anterior part of the nasal cavity is made up of septal cartilage [15]. Moreover, the nasal cavity sits below the ethmoid bone (cribriform plate) and above the connection site of the palatine bone and the palatine process of the maxilla, which is named the hard plate [16, 17]. The presence of variations in nasal cavity anatomy can affect airflow with minimal influence on its warming action. These anatomical variations are significantly observed in severe nasal septal deviations, giving rise to normal breathing disruption, snoring, sinusitis, and nasal obstruction [18].

## 3. Nasal Septal Deviation and Classifications

**Figure 2:**
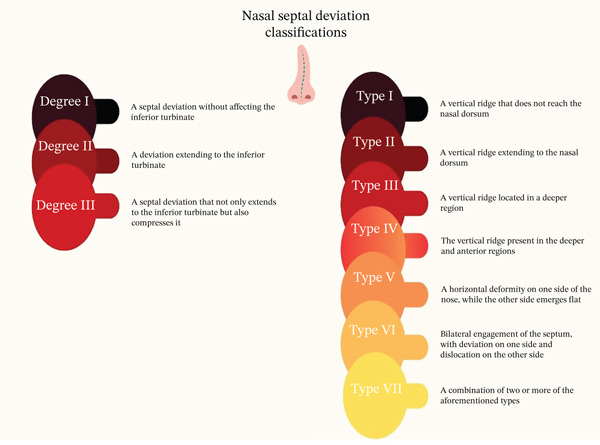
Classifications of nasal septal deviation.

Based on the extent of the deviation affecting the inferior turbinate, there is a classification for nasal septal deviation. To this end, three distinct degrees (Figure 2) have been defined for this classification [19].

1. Degree I: A septal deviation without affecting the inferior turbinate
2. Degree II: A deviation extending to the inferior turbinate
3. Degree III: A septal deviation that not only extends to the inferior turbinate but also compresses it [18, 19].

Another classification has been designed based on the prevalent deviation patterns, like S‐shaped and C‐shaped deviations. Mladina′s classification system has been offered to categorize NSD by relying on the observable features of the nasal septum through cone‐beam computed tomography (CBCT) or rhinoscopy. According to Mladina′s system, nasal septum deviation is categorized into seven different types as follows:.

Type I: A vertical ridge that does not reach the nasal dorsum.

Type II: A vertical ridge extending to the nasal dorsum.

Type III: A vertical ridge located in a deeper region.

Type IV: The vertical ridge present in the deeper and anterior regions.

Type V: A horizontal deformity on one side of the nose, whereas the other side emerges flat.

Type VI: Bilateral engagement of the septum, with deviation on one side and dislocation on the other side.

Type VII: A combination of two or more of the aforementioned types [20].

## 4. Diagnostic Tools for Deviated Nasal Septum

Currently, no standardized protocol has been suggested for diagnosing nasal septal deviation, which can largely justify the high incidence of this disorder. However, acoustic rhinometry (AR), nasal spectral sound analysis (NSSA), and rhinomanometry (RMM) are some diagnostic tests usable in assessing septal deviation [4]. AR utilizes the principle of acoustic reflection to measure nasal cavity geometry, such as nasal cavity volume (NCV) and nasal cross‐sectional area (NCA) in transient conditions related to breath‐holding, and is not influenced by subjective factors of the subjects [21]. NSSA is a cost‐effective and simple approach that apprises nasal airflow dynamically by evaluating the noise produced by turbulent airflow in the nasal cavity [22]. RMM offers a physiological evaluation of the nose dynamically by analyzing nasal airflow and transnasal pressure to estimate nasal resistance [23]. In addition to the mentioned methods, imaging, nasal endoscopy, and anterior rhinoscopy may be suitable modalities in the diagnosis of deviated nasal septum [22]. Imaging modalities, such as MRIs and CT scans, are able to detect a deviated nasal septum accurately in three dimensions. However, they are commonly exploited clinically to inspect paranasal conditions, like sinusitis, compared with an isolated deviated nasal septum [24, 25]. Nasal endoscopy and anterior rhinoscopy, once implemented in a decongested condition, can effectively identify the location and severity of a deviated nasal septum. However, it is a troubled procedure that is prone to considerable interrater variability [26, 27]. Overall, it has been declared that diagnostic methods like NSSA, RMM, and AR may be functional in detecting the deviated nasal septum in the anterior area of the nasal cavity. Nevertheless, these mentioned diagnostic tools, compared with imaging, nasal endoscopy, and anterior rhinoscopy, possess no specificity and sensitivity in detecting the existence, site, and severity of deviated nasal septum [4].

## 5. Septoplasty and Its Indications

Nasal septal deformity is considered the most common indication of septoplasty. This deviation is related to the bony and/or cartilaginous parts of the nasal septum, leading to a reduction in the cross‐sectional region, nasal blockage sensation, and disturbed airflow [28, 29]. Nasal blockage is one of the critical criteria for performing septoplasty. There are multiple scoring systems to classify the symptoms of nasal obstruction. The Nasal Obstruction Symptom Evaluation (NOSE) scale is known as a validated tool utilized to assess nasal obstruction levels. Subjects with low scores have a low chance of benefiting from septoplasty [30, 31]. Receiving a complete history to determine whether concomitant factors can participate or confer obstruction, like rhinosinusitis, trauma, vasculitis, allergies, malignancy, autoimmune disease, chronic use of decongestants, and illicit drug use, is crucial. In these situations, appropriate medical therapy, such as intranasal application of corticosteroids for chronic allergic rhinitis, should be assumed before proceeding to surgery [30]. Septoplasty may be required alongside skull base, endoscopic sinus, or orbital surgery to enhance access to desired structures [32, 33]. Other reasons for septoplasty comprise epistaxis (nosebleeds), chronic sinusitis, obstructive sleep apnea, and headaches or facial pains conferred by septal spurs irritating a turbinate (Sluder′s syndrome) [34].

## 6. Septoplasty and Contraindications

Some contraindications for septoplasty have been mentioned, such as concurrent diseases like vasculitis and rhinosinusitis or conditions in which medical therapy has not been completed [35]. Taking recreational medications, especially intranasal cocaine, during the perioperative period is not recommended. Cocaine′s vasoconstrictive and detrimental effects on the nasal mucosa can lead to complications, for instance, delayed healing, septal perforation, and eventually dorsal collapse, causing a saddle nose deformity [36]. Taking patient comorbidities, ASA grade, and age into account is critical to determining the safety of general anesthesia and whether patients can tolerate the recovery process postoperatively [35]. Patients who have unreal hope concerning the functional or aesthetic outcomes of septoplasty should not undergo surgery without thorough consultation preoperatively. This is especially important for subjects who also undergo rhinoplasty. The patient and surgeon need to have achievable expectations because this can elevate satisfaction for both parties following the procedure [37]. Moreover, individuals with septal deformity or deviation with restricted functional symptoms may experience minimal advantages from surgery [35].

## 7. Septoplasty and Its Complications

Subjects who underwent septoplasty may experience different complications. Hemorrhage/septal hematoma is one of the most prevalent complications after septoplasty. Based on reports, true hemorrhage following septoplasty is around 6%–13.4% of cases [38]. Another septoplasty complication that merits discussion is a cerebrospinal fluid (CSF) leak. This complication occurs when there is a tear in the dura mater, the protective membrane surrounding the brain and supporting structures of the skull base. Such a tear can create a connection between the nasal cavity and the subarachnoid space, leading to a leakage of fluid [39, 40]. Other complications encompass septal perforation, adhesions, structural deformity (i.e., nasal tip ptosis, nasal dorsum angulation, and saddle nose), anosmia/hyposmia, septal abscess, infection, toxic shock syndrome, endocranial complications (pneumocephalus), tooth anesthesia, and cardiac and ocular complications [38, 41]. The influences of each of these complications can be various. For example, infections may give rise to septal abscesses and endocranial conditions like septicemia or meningitis [42].

## 8. Updated Approaches to Elevate the Effectiveness of Septoplasty‐Related Approaches

### 8.1. Using the Temporalis Fascia and Silicone Films

Mainly to correct nasal septum deviations, nasal septal perforation (NSP) is expected following rhinological surgical strategies. Perforations are categorized into several types, including posterior and anterior; small, medium, and large; and symptomatic and asymptomatic. The conservative therapeutic approach involves the topical usage of moisturizing ointment and rinses with saline water. Among these, [43] have recommended a new, straightforward, and functional approach to repair symptomatic NSP using a temporalis fascia graft and long‐term protection with silicone sheets. Overall, the surgical procedure was performed in six phases, including anesthesia and early access (Phase 1), exposure and preparation (Phase 2), graft collection (Phase 3), graft placement (Phase 4), long‐term protection (Phase 5), and closure (Phase 6). The surgery was accomplished under general anesthesia. The surgeon initiated with an open approach, making an inverted V‐shaped incision on the columella (the tissue between the nostrils). In the next step, the nasal septum was exposed by carefully dissecting above the perichondrial layer. The scar tissue from a prior surgery made it difficult to separate the mucosal layers around the perforation; however, this was carried out to create enough space for the graft. A graft was obtained from the temporalis fascia of the own patient (removed from the right side of the subjects). The graft was trimmed to be slightly larger than the septal perforation to compensate for any shrinkage in the future.

The fascia graft was placed in the space created between the two septal mucosal flaps. First, it was secured with absorbable sutures to the right‐side flap. Then, the graft was stretched to completely cover the defect. A critical point of the technique was that the mucosal flaps were not sutured closed over the graft so that the restorative process could be performed based on mucosal migration across the graft surface. To long‐term protect in this strategy, two thin silicone sheets, one on each side of the septum, were placed. These sheets were sutured in place and left for at least 6 weeks. This shield protected the graft from air turbulence and physical disruption while allowing direct monitoring of the restorative process. In the end, the initial columella incision was closed without using traditional nasal packing such as a tamponade. This method was applied to 10 subjects with symptomatic NSP. After a year of follow‐up, all cases but one benefited from successful outcomes, with complete defect closure, revealing a 90% success rate. In the only unsuccessful case, a large perforation (> 3 cm) was presented. In this patient, a small recurrence (< 5 mm) was observed at the back edge of the repair site 4 months after the operation. The temporalis fascia graft was successfully inserted in all patients without serious complications. The prolonged placement of silicone sheets was well‐tolerated, with only controllable minor crusting observed. Moreover, all patients experienced considerable symptomatic improvement, encompassing restored nasal breathing and the cessation of recurrent epistaxis and crust formation [43]. It is worth noting that this study was small, single‐centered, and lacked a control group. Therefore, the promising success rate requires validation through larger, randomized controlled trials to establish its generalizability. Taken as a whole, this research accentuated the effectiveness of the described approach for repairing symptomatic NSP. The authors revealed that the effectiveness is not related to the complexity of this surgical technique, but to the long‐term safeguarding of the surgical site. In this work, the silicone sheets created an ideal space for mucosal regrowth by isolating the graft from the traumatizing airflow.

### 8.2. Platelet‐Rich Plasma (PRP)

PRP is a plasma fraction rich in bioactive molecules and growth factors derived from anticoagulated autologous blood that has an important role in regulating inflammation and promoting tissue regeneration [44, 45].

**Figure 3:**
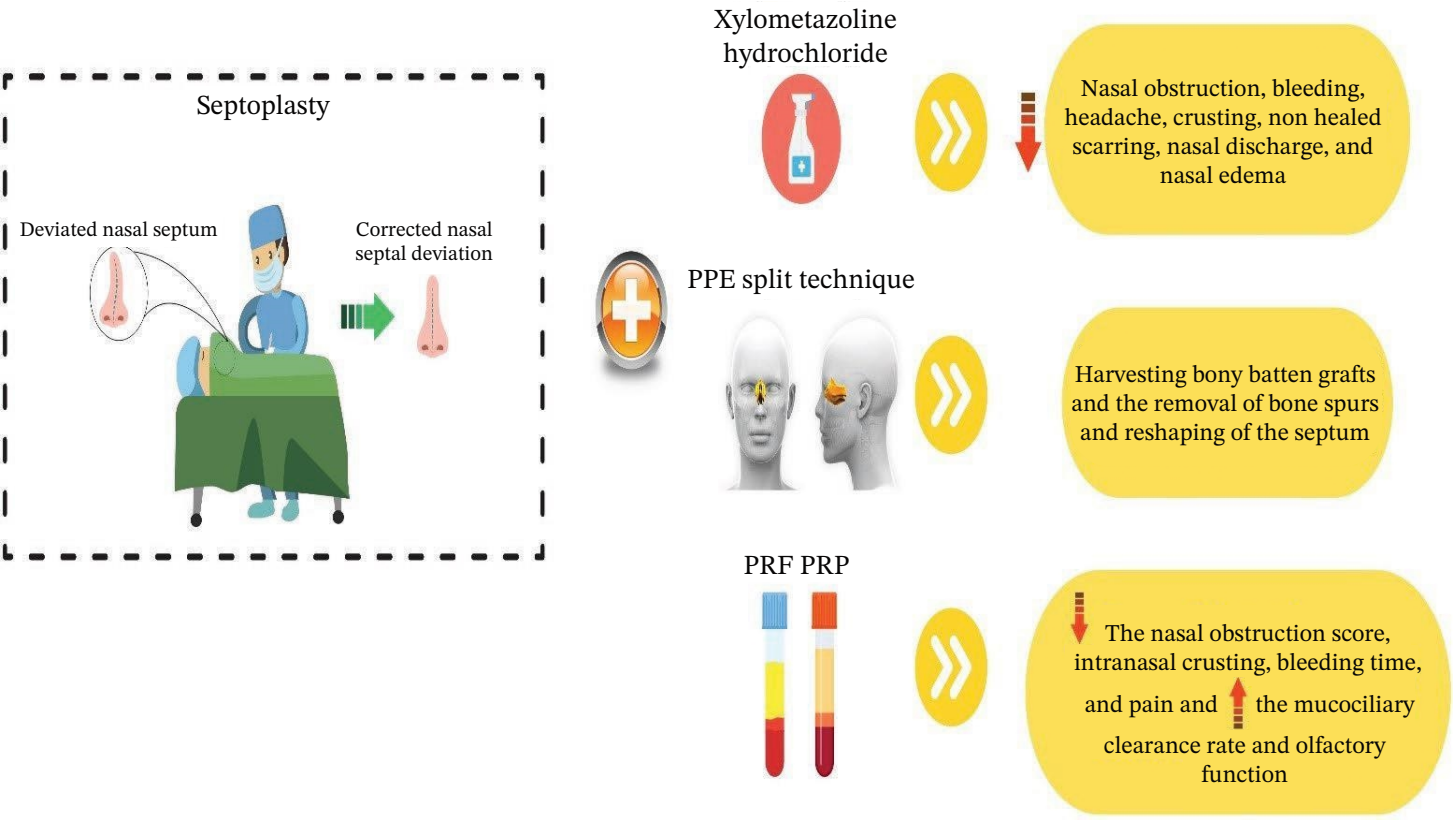
Some proposed approaches to elevate the effectiveness of septoplasty.

In a prospective randomized observational study, the effectiveness of submucoperichondrial utilization of autologous PRP on nasal mucosal healing following septoplasty was inspected (Figure 3). This research was performed on 40 cases that experienced septoplasty surgery to correct septal deviations. In the PRP group (*n* = 21), this type of plasma, prepared from the patient′s own blood, was applied to the entire septal mucosal surface and injected into the submucoperichondrial/submucoperiosteal space. Mucociliary clearance time (saccharin test), nasal obstruction score (visual analogue scale [VAS]), bleeding times, and the existence of nasal crusting were assessed at three time points with a time interval of 5 days following surgery, and the results were compared between the PRP group and the control group (*n* = 19), receiving saline solution in the same areas.

On Day 10, nasal crusting was remarkably lower in the PRP group compared with the control group (33.3% vs. 68.4%). Also, nasal obstruction scores reported by patients were significantly lower in the PRP group on both Days 10 and 15. Although not statistically significant, the PRP group displayed a trend towards decreased bleeding time, faster mucociliary clearance, and a lower incidence of mucosal lacerations, giving rise to septal perforation in comparison with the control group. In conclusion, it was found that PRP can have a constructive role in nasal mucosal repair, congestion, and nasal crusting following septoplasty [46].

### 8.3. Platelet‐Rich Fibrin (PRF)

PRF is known as a second‐generation platelet‐related product that enhances wound healing by increasing growth factors and fibroblasts [47]. Interestingly, there is evidence showing that administration of PRF to the mucosal surface of patients who underwent septoplasty improves pain score and olfactory function, particularly in the initial postoperative period. These findings have been reported in the project of Tutar et al.

In this prospective, randomized, observational study, 141 participants who underwent septoplasty were included and divided into two groups: Group 1 (*n* = 74), cases who were given PRF therapy on the nasal mucosal surface following the septoplasty, and Group 2 (*n* = 67), cases who underwent septoplasty and did not receive PRF therapy. Patient olfactory function was assessed using the Sniffin′ Sticks test, which addresses a composite threshold, discrimination, and identification (TDI) score. Assessments were performed preoperatively and during follow‐up at 1 week, 6 weeks, and 6 months after surgery, with patients classified based on their scores as normosmic, hyposmic, or anosmic. Simultaneously, postsurgery pain levels were controlled using a VAS at Weeks 1 and 3. At the first stage of the surgery, a hemitransfixion incision was made on the side of the nasal septal deviation. The bilateral septal mucosal flap was then raised, and the cartilage and the deviated septal bone were removed. Afterward, the obtained PRF was settled on the upper part of the septal mucosa to support the olfactory epithelium [48]. PRF was produced from 20 mL of autologous blood by centrifuging it without an anticoagulant and subsequently collecting the fibrin clot from the intermediate layer. The attained results divulged that PRF therapy can confer significant early postoperative advantages. Before surgery, olfactory function was similar between the two mentioned groups. However, a significant difference was reported 1 week postsurgery. At this time, a conspicuously higher percentage of patients in the PRF group (89.2%) maintained normal smell function (normosmia) compared with the non‐PRF group (73.1%), which also experienced cases of hyposmia and even anosmia. This usefulness was temporary; by the 6‐week and 6‐month follow‐ups, olfactory function had improved and normalized in both groups, with no significant differences remaining. Plus, subjects in the PRF group declared significantly lower pain levels at both 1 week and 3 weeks after surgery, revealing that PRF not only aids in preserving the sense of smell but also effectively reduces postoperative discomfort during the initial healing stage.

### 8.4. Using PRP and Fascia Lata Graft

**Figure 4:**
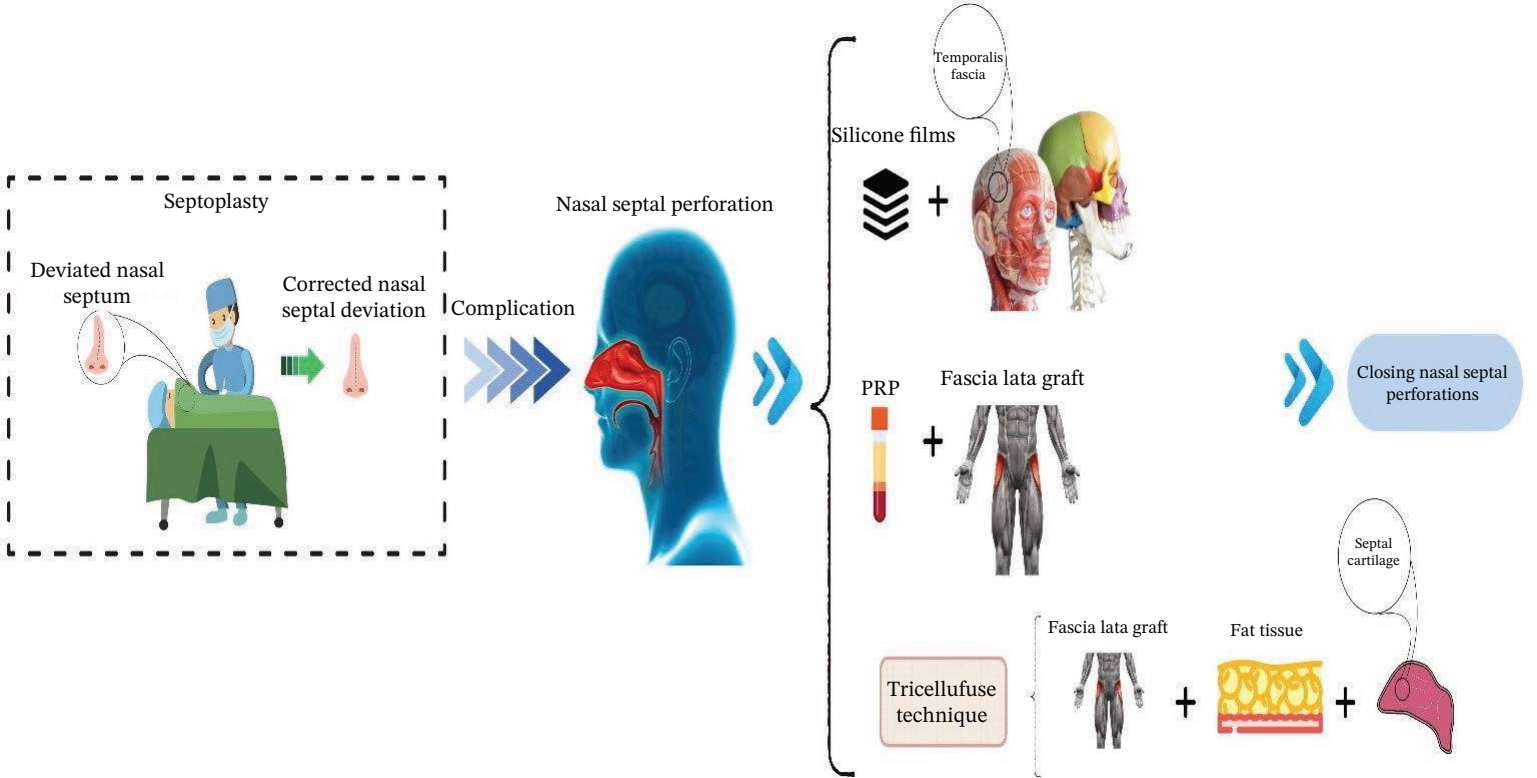
Some proposed approaches to resolve one of the most common complications of septoplasty.

Hanci et al. have recently tested a surgical technique based on a combination of fascia lata graft and PRP to repair NSP in 25 cases who underwent NSP repair (Figure 4).

In this retrospective study, for better visibility, an open rhinoplasty technique was applied. In this research, the dorsal nasal skin was lifted to access the nasal septum between the medial crura of the lower lateral cartilages. In the next step, bilateral mucoperichondrial flaps were elevated to cover the perforation in a complete 360° fashion. Subsequently, a fascia lata graft was obtained from the patient′s right thigh. On the other hand, PRP was harvested from the patient′s autologous venous blood during surgery through a centrifugation process. The PRP was placed on the fascia lata, which was subsequently folded and sutured to encapsulate it, leading to the creation of a fusion fascia lata graft (FFLG). The perforation edges were prepared, and the FFLG was mildly inserted via the mucoperichondrial flaps in order to cover the perforation. Postoperative care included the administration of nasal splints for 1 month and antibiotics and moisturizing nasal sprays to help with healing. To evaluate the success rate, patients were assessed at 1 and 12 months after surgery. The mentioned researchers have declared that the complete closure of the perforation was performed on 23 cases out of 25 (92% success rate). Successful closure was observed in all large perforations (*n* = 17). However, two medium‐sized perforations (1.5 and 1.7 cm) failed to close; both of these cases were in smokers. The retrospective nature of this study, along with its modest sample size, restricts the strength of the conclusions drawn. Besides, the fact that both failures occurred exclusively among smokers emphasizes the significance of patient factors. This suggests that the efficacy of the technique may depend on the specific population being studied [49].

### 8.5. Tricellufuse Technique

A team of researchers has evaluated the performance of the TriCelluFuse technique, using a graft consisting of three types of the patient′s own tissue, diced septal cartilage, fat tissue, and fascia lata, to close NSP in medium‐sized (0.5–2 cm). In a retrospective analysis of 22 subjects undergoing NSP repair, the surgical procedure was conducted using an open rhinoplasty approach. The operation began with a Goodman incision combined with alar rim incisions, followed by the elevation of the dorsal nasal skin to access the septum. Bilateral mucoperichondrial flaps were then raised to ensure 360° coverage of the perforation.

The innovative step of the procedure was the creation of the TriCelluFuse graft. Through a single incision on the patient′s right thigh, both the fascia lata tissue and underlying fat were harvested. Meanwhile, cartilage was obtained from the patient′s own deviated nasal septum and finely diced. The fat was processed into microfat and, together with the diced cartilage, was placed onto the spread fascia lata. This assembly was then folded and sutured with 4.0 Vicryl material to create a single, multilayered graft.

This composite graft was gently inserted through the mucoperichondrial flaps to cover the perforation. Postoperative care included nasal splinting for 1 month, a course of antibiotics, and the use of moisturizing sprays. Patients were monitored closely, with follow‐up examinations at 1, 2, 6, and 12 months.

The results manifested that the mentioned technique was highly effective, achieving complete closure of NSP in 19 out of 22 patients, which corresponds to a success rate of 86.3%. A critical finding was that all three unsuccessful cases, where closure was not observed after 1 year, occurred in smokers, with statistical analysis confirming a strong link between smoking and surgical failure. This underscores smoking as a significant risk factor, especially when contrasted with the 100% success rate achieved in nonsmoking patients. Furthermore, no complications were reported at the thigh donor site. Based on these outcomes, the researchers concluded that the technique is a safe and reliable solution for repairing medium‐sized septal perforations [50]. Although the success rate in this study was impressive, the strong correlation with smoking status suggests that the technique′s true efficacy is best evaluated in a controlled, prospective setting that can consider such confounding variables. The findings are encouraging, but still preliminary.

### 8.6. Perpendicular Plate of Ethmoid (PPE) Split Technique

The ethmoid bone is one of the best grafts for supporting the nasal septum L‐strut compared with other graft options [51]. In a scientific project, the PPE split technique has been suggested for the effective resection of the bony septum or the collection of partial PPE for use in septal batten grafts in septoplasty and rhinoplasty, especially in subjects with thick PPE.

This retrospective study by Kyung Won Kwon, involving 36 patients (22 septoplasties and 14 rhinoplasties), inspected a novel “PPE split” technique. The key purpose of this modality was to minimize septal defects and prevent complications while successfully harvesting bony grafts of favorable thickness. The procedure comprised detaching the PPE from the septal cartilage and performing a vertical osteotomy with a No. 10 scalpel blade. This technique was utilized for two main goals: harvesting bony batten grafts to provide structural support and for the removal of bony spurs to reshape the septum. The outcomes pointed out the technique′s overall effectiveness, with successful PPE harvesting achieved in the vast majority of patients. However, graft failure was reported in three instances during batten graft harvesting. Despite this, the study confirmed the validity and effectiveness of the PPE split technique as a valuable supplementary approach in septoplasty and rhinoplasty, accentuating its ability to achieve surgical aims while preserving septal integrity [52].

### 8.7. Xylometazoline‐Hydrochloride Nasal Decongestant Spray

Xylometazoline hydrochloride, a potent alpha‐adrenergic agonist, serves as a nasal decongestant by constricting nasal blood vessels, thereby reducing mucosal swelling and improving airflow [53]. Although its use in routine nasal congestion is common, its application following septoplasty is a subject of debate due to concerns about rebound congestion (rhinitis medicamentosa) upon discontinuation and potential impairment of mucosal healing due to reduced blood flow. Nonetheless, a recent triple‐blinded randomized clinical trial by [54] investigated the efficacy of a 0.1% xylometazoline hydrochloride spray compared with a 0.9% saline spray for postseptoplasty symptom relief. In this study, patients using the xylometazoline spray for 1 week showed significantly lower rates of nasal obstruction, bleeding, and edema, as well as improved endoscopic findings on postoperative days 3 and 7, resulting in higher patient satisfaction with no reported adverse effects during the study period. The authors concluded that short‐term use was highly effective. However, these positive findings should be interpreted with consideration of the noted controversies, particularly the risk of rebound effects beyond the 1‐week study duration. This suggests that, although potentially effective, the technique requires further long‐term study and careful patient selection for consideration in clinical use [54].

### 8.8. Evidence From Large‐Scale and Multicenter Trials

In a multicenter clinical trial performed by Carrie et al. (2023), the efficacy of septoplasty compared with a standardized treatment plan was scrutinized in adults suffering from nasal obstruction because of a deviated nasal septum across 17 hospitals in the United Kingdom [55]. Three hundred seventy‐eight cases (≥ 18) with septal deviation and remarked nasal obstruction (NOSE score ≥ 30) admitted to the ENT clinics were allocated into two groups, including the septoplasty (*n* = 188) and medical (*n* = 190) plan groups. In the surgical intervention group, patients underwent septoplasty (with or without inferior turbinate reduction) following 12 weeks of randomization. In another group, volunteers received a 6‐month regimen of two types of nasal sprays, including mometasone furoate and isotonic saline. After 6 months, subjects undergoing septoplasty revealed a 20‐point greater enhancement on the SNOT‐22 (22‐item Sinonasal Outcome Test) symptom scale compared with those managed with the medical plan. The observed difference was statistically significant (95% CI: −23.6 to −16.4; *p* < 0.001) and clinically important [55]. The clinical advantages of septoplasty were maintained at 1 year. Applied surgical intervention caused a dramatic improvement in all subdomains related to SNOT‐22 (sleep, nasal, psychological, and ear/facial), clinical measurement of nasal airflow, and total quality of life. Also, a subpopulation treatment effect pattern plot (STEPP) analysis indicated increased benefits of septoplasty with baseline severity. Cases with the most severe obstruction before operation (NOSE scores ~80–100) showed the greatest improvement (~30 points on SNOT‐22). In terms of safety, the surgery imposed some complications, comprising infection needing antibiotics (12%), a changed sense of smell (11%), and bleeding needing readmission following the operation (4%). On the whole, this large‐scale research highlighted substantial, long‐lasting, and considerable benefits of septoplasty for patients with the most severe symptoms of nasal septum deviation [55]. Another large‐scale study was conducted in 18 hospitals in the Netherlands by Egmond et al. In this clinical project, 203 participants (≥ 18 years) were assigned to the surgical (septoplasty within 6–8 weeks; *n* = 102) and nonsurgical (routine nonsurgical care or medical treatment [typically local corticosteroids]; *n* = 101) groups. The assessed primary outcome in this work was Health‐related quality of life evaluated by the Glasgow Health Status Inventory (GHSI). Secondary outcomes comprised disease‐specific indices (NOSE scale and SNOT‐22), objective nasal patency (peak nasal inspiratory flow [PNIF], general life quality (EQ‐5D‐3L), and 4‐phase rhinomanometry [4PR]), and complications. In the surgical arm, the mean score of GHSI was significantly higher (72.2 vs. 63.9; mean difference 8.3, 95% CI 4.5–12.1), revealing improved life quality. The secondary outcomes potentiated the primary findings, addressing remarkable benefits for the surgery in terms of both subjective and objective indices. Nasal‐specific symptom scores pointed out significant improvements, as the SNOT‐22 and NOSE scales revealed mean differences of 9.7 and 17.8 points in favor of septoplasty, respectively. Objective assays confirmed improved airflow in surgical patients, with a significant 29.3 L/min increase in PNIF, despite more variable results from 4PR. Plus, the positive Glasgow Benefit Inventory score of 10.6 displayed an obvious benefit for surgical patients from their treatment, whereas a general quality of life measure (EQ‐5D‐3L) did not change compared with the surgical group. Also, nonserious complications, like infection and hemorrhage, following septoplasty, occurred in about 20% of surgical cases. Serious problems such as septal perforation and abscess were observed in 2 and 1 cases, respectively. No detrimental influences arising from nasal medications were documented. Collectively, this study exemplified that septoplasty is considerably more effective than nonsurgical management in order to ameliorate life quality and nasal obstruction in adult individuals with a deviated septum, and its benefits are maintained for at least 2 years. These clinical trials provide high‐quality evidence to validate septoplasty as a favorable intervention, showing previous clinical uncertainty and informing future guidelines [56].

## 9. Conclusion

Nasal septal deviation, as the leading cause of nasal airway obstruction, can cause various problems, like breathing problems, sinus infections, exacerbating sleep apnea, and sleep disturbances. Therefore, patients who suffer from nasal septal deviation need appropriate intervention to prevent the mentioned consequences. Currently, septoplasty surgery is one of the most suitable techniques for correcting nasal septal deviation in related patients. Nevertheless, this approach still demands remarkable advancements to improve its efficacy and lower or resolve septoplasty‐associated complications, especially NSP and the recurrence of deviation in adolescents and adults. Recently, significant efforts have been made to enhance the clinical effectiveness of septoplasty while minimizing associated health challenges. Recent studies present a mixed view of the evidence surrounding septoplasty. Although large, multicenter trials have strongly addressed the overall effectiveness of the procedure, newer techniques—such as PRP, PRF, and TriCelluFuse grafts—show early promise for potentiating healing and preventing perforations. However, these innovative strategies are currently only supported by small, preliminary studies and lack the strong evidence that backs the standard procedure. Thus, their widespread use should await confirmation from future large‐scale, comparative research.

## Author Contributions

M.J.: conceptualization, data curation, formal analysis, methodology, and writing—original draft; F.R‐T.: conceptualization, methodology, writing—review and editing, supervision, and validation.

## Funding

No funding was received for this manuscript.

## Ethics Statement

This is a review article. Hence, ethical approval was not required for the study.

## Consent

The authors have nothing to report.

## Conflicts of Interest

The authors declare no conflicts of interest.

## Data Availability

The data that support the findings of this study are available from the corresponding author upon reasonable request.
